# The Air We Breathe

**Published:** 1875-02

**Authors:** 


THE AIR WE BREATHE.—A comparison of the losses of heat by the respiration of an absolutely dry and absolutely saturated air, shows that at 32 deg. and dry, we lose 1,172 caloric units; and at 86 deg. and dry, we lose 1,096 caloric units—a difference of only 76; but at 32 deg. and saturated, we lose 1,060 caloric units; and at 86 deg. and saturated, 420 caloric units—a difference of as much as 640 caloric units. The different states of dryness of the air appear thus to be of greater moment than the difference of temperature, and this is the reason why our sensations do not always coincide with the thermometer. The working power of the body depends upon a certain amount of consumption, by which a certain amount of heat is necessarily created, and which has to leave the body in a regular way. The Hindoo who has to draw the European's punkah bears the heat better in proportion, as he takes less food and creates less heat in himself, but then his working power is quite proportionate to the total of his consumption. The European's struggle in a hot climate, and his dangers of degeneracy, will remain as long as he has no better means of cooling himself—say some contrivance by which the air in the house could be deprived of water.

---

If women want warm feet they must give up wearing garters around the leg. The stockings should be suspended by straps from the waist.

